# Cullin 3/with No Lysine [K] Kinase/Ste20/SPS-Related Proline Alanine Rich Kinase Signaling: Impact on NaCl Cotransporter Activity in BP Regulation

**DOI:** 10.34067/KID.0000000000000527

**Published:** 2024-08-09

**Authors:** Kingsley Omage, James A. McCormick

**Affiliations:** Division of Nephrology and Hypertension, Department of Medicine, Oregon Health and Science University, Portland, Oregon

**Keywords:** BP, cell and transport physiology, cell signaling, hypertension, kinase, sodium (Na^+^) transport

## Abstract

The sodium chloride cotransporter (NCC) fine-tunes Na^+^ balance and indirectly affects the homeostasis of other ions including K^+^, Mg^2+^, and Ca^2+^. Owing to its effects on Na^+^ balance, BP is significantly affected by alterations in NCC activity. Several factors have been reported to influence the expression and activity of NCC. One critical factor is NCC phosphorylation/dephosphorylation that occurs at key serine-threonine amino acid residues of the protein. Phosphorylation, which results in increased NCC activity, is mediated by the with no lysine [K] (WNK)-SPS–related proline alanine rich kinase (SPAK)/OSR1 kinases. NCC activation stimulates reabsorption of Na^+^, increasing extracellular fluid volume and hence BP. On the other hand, proteasomal degradation of WNK kinases after ubiquitination by the Cullin 3-Kelch-like 3 E3 ubiquitin ligase complex and dephosphorylation pathways oppose WNK-SPAK/OSR1-mediated NCC activation. Components of the Cullin 3/Kelch-like 3–WNK-SPAK/OSR1 regulatory pathway may be targets for novel antihypertensive drugs. In this review, we outline the impact of these regulators on the activity of NCC and the consequent effect on BP.

## Introduction

Hypertension is considered primary or essential when the cause of elevated BP is not easily understood or secondary when the cause is due to medical conditions like diseases of the renovascular system, drug-induced hypertension, or diseases of the endocrine system. Primary hypertension likely emerges from intricate interactions between the surrounding environment and the genetic makeup of the individual. The prevalence of hypertension in adults in the United States, regardless of cause, is estimated to be >45%.^1^ The importance of the NaCl cotransporter (NCC) expressed along the distal convoluted tubule (DCT) in BP regulation is demonstrated by the efficacy of thiazide diuretics, which directly block the ability of NCC to reabsorb NaCl, as a first-line antihypertensive therapy.^2^ Mutations in genes that encode components of the Cullin 3/with no lysine [K] (WNK)/STE20/SPS1-related proline alanine rich kinase (SPAK) pathway that regulates NCC cause the rare monogenic disease familial hyperkalemic hypertension (FHHt, also known as Gordon syndrome or pseudohypoaldosteronism type 2).^3–5^ The importance of this pathway in humans is illustrated not only by the rare disease FHHt but also by the association of variants in Cullin 3 (*CUL3*),^6^ Kelch-like 3 (*KLHL3*),^7^ *STK39* (SPAK),^8,9^ *WNK1*,^10,11^ and *WNK4*^12^ with human hypertension. The Cullin 3/WNK/SPAK pathway ultimately affects the phosphorylation status of NCC, which determines NCC activity. Therefore, a good understanding of the regulatory mechanisms of the NCC will not only provide insight into the regulation of BP but also identify potential therapeutic interventions for hypertension. This review outlines the regulatory impact of the Cullin 3/WNK/SPAK pathway on the activity of NCC and how it affects BP.

## Activation of NCC by Protein Kinases

**Figure 1:**
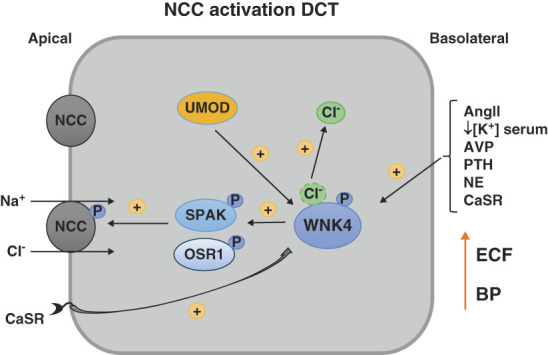
**Mechanisms that increase NCC activation.** The NaCl cotransporter (NCC) expressed along the DCT is activated by phosphorylation mediated by the WNK4-SPAK/OSR1 pathway. WNK4 is activated by several mediators at the basolateral pathway including AngII, lower serum [K^+^], AVP, PTH, NE, and the CaSR, which also mediates WNK4 activation *via* effects at the apical membrane. Intracellular UMOD may also activate WNK4 signaling. WNK4 is inhibited by direct binding of Cl^−^; Cl^−^ dissociation promotes WNK4 autophosphorylation and activation. NCC activation increases ECF volume (ECF) and thus BP. AngII, angiotensin II; AVP, arginine vasopressin; CaSR, calcium-sensing receptor; DCT, distal convoluted tubule; ECF, extracellular fluid; PTH, parathyroid hormone; SPAK, SPS-related proline alanine rich kinase; UMOD, uromodulin; WNK, with no lysine [K].

**Figure 2:**
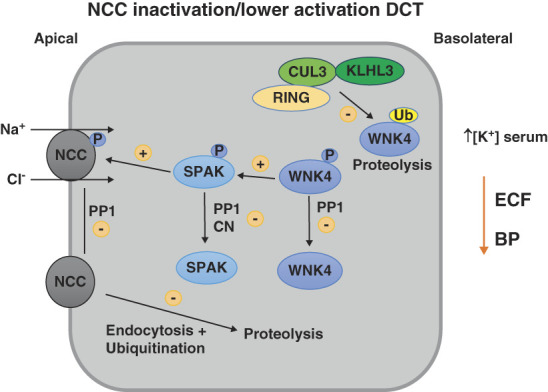
**Mechanisms that lower NCC activity**. Activity of the NaCl cotransporter (NCC) along the DCT is reduced by dephosphorylation either directly or indirectly. PP1 may directly dephosphorylate NCC and may also lower activation of NCC by dephosphorylating the protein kinases SPAK, OSR1 (not shown), and WNK4 that mediate NCC phosphorylation. CN also promotes dephosphorylation of SPAK and NCC, reducing NCC activation. Dephosphorylation of NCC stimulates its endocytosis from the apical membrane, promoting NCC ubiquitination and proteasome-mediated proteolysis, lowering NCC levels in the cell. A ubiquitin ligase complex consisting of CUL3, KLHL3, and a RING ubiquitin ligase tags WNK4 for proteolysis by the proteasome, lowering NCC activation. WNK4 ubiquitination can be reduced *via* angiotensin II–mediated phosphorylation of KLHL3 (not shown), promoting NCC activation. Higher serum [K^+^] inhibits NCC phosphorylation by leading to higher intracellular [Cl^−^] (not shown), which inhibits WNK4 autophosphorylation (see Figure 1). NCC inhibition lowers ECF volume (ECF) and thus BP. CN, calcineurin; CUL3, Cullin 3; KLHL3, Kelch-like 3; PP1, protein phosphatase 1; RING, really interesting new gene.

Active NCC is located at the apical membrane of the DCT and transports Na^+^ and Cl^−^ into the cell from the tubular lumen. It is highly sensitive to thiazide diuretics and is tightly regulated physiologically. One critical function is in fine-tuning the reabsorption of Na^+^ along the DCT, but its activity also plays a key role in K^+^, Mg^2+^, and Ca^2+^, and HCO_3_^−^ homeostasis through indirect effects on not only the DCT but also on other tubule segments. Phosphorylation of NCC at several amino acid residues along its amino terminus (Thr46, Thr55, and Thr60) not only activates the transporter^13^ (Figure 1) but also blocks NCC endocytosis, thus stabilizing it in the apical membrane^14,15^ (Figure 2).

Much of our understanding of NCC regulation comes from the identification of mutations that cause FHHt, a rare disorder characterized by hyperkalemia early in life, and later onset hypertension. The renal manifestations of FHHt are primarily due to increased NCC activity because thiazide diuretics reverse the electrolyte and BP abnormalities, although in more severe forms, there is a vascular component. The first FHHt causative genes identified were two members of the WNK kinase family, *WNK1* and *WNK4*.^5^ Mutations in these genes ultimately increase the abundance of WNK1 or WNK4 along the DCT. However, WNK kinases do not directly phosphorylate NCC (Figure 1). Rather, NCC is primarily phosphorylated by SPAK, with some contribution of the kinase oxidative stress responsive kinase-1 (OSR1).^13^ WNK kinases fit into the pathway by phosphorylating SPAK and OSR1,^16,17^ with the activated SPAK and OSR1 then phosphorylating NCC by binding directly to it.^13^ Mouse models have refined our understanding of the pathway and shown that WNK1 and WNK4, and SPAK and OSR1, are not equal *in vivo*.

## Mouse Models with WNK-SPAK/OSR1 Disruption

Knockout mouse models have been generated for each component of the WNK-SPAK/OSR1 pathway, and their phenotypes have revealed differential contributions of each kinase to NCC activation *in vivo*, despite displaying similar effects to promote NCC activation *in vitro*. SPAK (*Stk39*) knockout mice display a dramatic reduction in NCC phosphorylation (>90%) accompanied by a reduction in BP that was exacerbated by dietary salt restriction.^18–20^ By contrast, while OSR1 (*Oxsr1*) knockout mice displayed lower BP than wild-type mice, this was associated with lower phosphorylation of the furosemide-sensitive Na^+^-K^+^-2Cl^−^ cotransporter, expressed along the thick ascending limb (TAL) of the Loop of Henle rather than loss of NCC phosphorylation.^21^ Mice with disruption of both SPAK and OSR1 in kidney (SPAK/OSR1 double knockout mice) display a further reduction in NCC phosphorylation from that seen in SPAK knockout mice suggesting a role, albeit minor, for OSR1 in NCC regulation *in vivo*.^22^

WNK1 (*Wnk1*) knockout mice die during development because of an essential role for WNK1 in development of the vasculature.^23,24^ However, WNK1 has not yet been knocked out specifically along the renal tubule or DCT, so its precise roles in the activation of NCC are not known. While mice heterozygous for WNK1, with one allele disrupted, display a slight reduction in BP, this may reflect effects on vascular tone.^24,25^ Mouse models with overexpression of WNK1 display higher NCC phosphorylation and BP, ^26^ suggesting WNK1 can activate NCC *in vivo*. However, this model may not reflect normal physiology because it was generated by introducing a *WNK1* mutation that causes FHHt. These *WNK1* mutations are located in regulatory sequences of introns in the gene and lead to ectopic WNK1 expression along DCT.^27^

The *WNK1* gene also encodes a shorter form of WNK1 that lacks the kinase domain, called kidney-specific WNK1 (KS-WNK1) because of its high expression in kidney, and more specifically, along the DCT.^28,29^ KS-WNK1 was originally believed to act as an inhibitor of full-length kinase active WNK isoforms. However, data from mice lacking KS-WNK1 made by different groups have produced conflicting data,^30–32^ with more recent data suggesting that KS-WNK1 promotes activation of WNK-SPAK/OSR1 signaling by facilitating assembly of protein complexes called WNK bodies.^33,34^

Characterization of WNK4 (*Wnk4*) knockout mice has revealed that WNK4 rather than WNK1 is the critical WNK kinase for NCC activation *in vivo*. The phenotype of WNK4 knockout mice closely resembles that of SPAK knockout mice, with almost complete loss of NCC phosphorylation and activity.^35^ WNK4 knockout mice displayed normal BP at baseline but lower BP when placed on a salt-restricted diet. Note that the phenotypes of both WNK4 and SPAK knockout mice closely resemble the human disorder Gitelman syndrome, caused by inactivating mutations in *SLC12A3* encoding NCC, and recapitulated in mouse models carrying Gitelman syndrome mutations.^15,36^ In each case, there is loss of NCC activity accompanied by hypokalemia and metabolic alkalosis. Furthermore, BP is normal or low, with salt restriction lowering BP.

## The WNK-SPAK/OSR1 Pathway Is Modulated by Ubiquitination

While WNK kinase mutations were shown to cause FHHt in 2001, it was only in 2012 that the causative mutations for the majority of FHHt cases were identified, mutations in the *CUL3* and *KLHL3* genes. These genes encode Cullin 3 and Kelch-like 3, components of a ubiquitin ligase complex that transfers the small protein ubiquitin onto proteins, often targeting the protein for degradation by the proteasome. *In vitro* studies demonstrated that WNK kinases, but not SPAK, OSR1, or NCC, are targets of the CUL3-KLHL3 E3 ubiquitin ligase complex^37,38^ (Figure 2). Thus, CUL3-KLHL3 activity lowers WNK-SPAK/OSR1 signaling by reducing WNK kinase abundance. Mutations in CUL3, KLHL3, or WNK4 prevent assembly and normal function of the ubiquitination complex,^39^ leading to accumulation of WNK4 and perhaps WNK1. This causes FHHt by increasing activation of SPAK and hence NCC. KLHL3 is expressed at extremely high levels along the DCT compared with other tubule segments,^40^ and KLHL3 (*Klhl3*) knockout mice recapitulate the FHHt phenotype, with hyperkalemia and hypertension.^41^ By contrast, CUL3 (*Cul3*) knockout mice have a complex phenotype featuring renal fibrosis, salt-sensitive hypotension, and polyuria due to loss of CUL3 protein expression along the entire tubule.^40,42^ The mechanism by which *CUL3* mutations cause FHHt is therefore more complex than a simple loss of function,^39^ but mice expressing the mutant CUL3 protein clearly display an FHHt phenotype.^43–45^ The mutant CUL3 protein displays many defects including higher affinity for KLHL3, and it also promotes degradation of itself and KLHL3.^39^ These defects impair formation of the CUL3-KLHL3 ubiquitin ligase complex, preventing WNK kinase ubiquitination and ultimately increasing NCC activation by SPAK. *CUL3* mutations cause the most severe form of FHHt, with earlier onset of hypertension than is seen with *WNK1*, *WNK4*, or *KLHL3* mutations.^3,46^ This is likely due to an important role for CUL3 in controlling vascular tone, a function dysregulated when mutant CUL3 is expressed.^47,48^

## Physiological Regulation of NCC

NCC activity is modulated by multiple mechanisms, including the classic renin-angiotensin-aldosterone system that is stimulated in response to ECF volume contraction. Many of these mechanisms involve signaling through the WNK-SPAK/OSR1 pathway.

### Angiotensin II and Aldosterone

There is some evidence that angiotensin II (AngII) can directly stimulate NCC activity. Acute administration of AngII to rats increases NCC abundance at the apical membrane of the DCT,^49^ and NCC phosphorylation increased in adrenalectomized rats after AngII infusion.^50^ These studies suggest an aldosterone-independent effect of AngII to activate NCC. San-Cristobal and colleagues showed that in the *in vitro Xenopus laevis* oocyte expression system, activation of NCC by AngII occurs *via* a mechanism that depends on the AT1R-WNK4-SPAK pathway.^51^ Furthermore, in contrast to the activating effect observed in wild-type mice, AngII infusion failed to stimulate phosphorylation of SPAK and NCC in WNK4 knockout mice.^52^ This indicates that angiotensin-dependent activation of NCC requires an intact WNK/SPAK/OSR1 signaling pathway. AngII may also promote NCC phosphorylation by inducing phosphorylation of the WNK4 binding region of KLHL3, impairing the ability of WNK4 to bind CUL3-KLHL3 and thus reducing WNK4 ubiquitination and subsequent proteasomal degradation.^53,54^ The increase in WNK4 levels would then promote NCC phosphorylation through SPAK/OSR1.

Dietary salt restriction or chronic infusion of aldosterone has been reported to lead to increased abundance of phosphorylated and total NCC.^55^ Aldosterone-induced NCC activation is also associated with increased phosphorylation of SPAK^56^ and increased phosphorylation of NCC, which results from an acute effect in the DCT.^57^ However, studies in mineralocorticoid receptor knockout mice suggest that effects of aldosterone on NCC are secondary to effects on serum [K^+^].^58,59^ These mice displayed salt-wasting, low BP, hyperkalemia, and lower NCC phosphorylation, but dietary K^+^ restriction increased NCC phosphorylation and corrected the electrolyte abnormalities and BP defect.^58,59^

### Dietary Potassium Intake

It is now well established that NCC phosphorylation and activity are inversely correlated with serum [K^+^].^60^ The degree of NCC activation serves as a homeostatic mechanism to maintain serum [K^+^] by metering delivery of Na^+^ from the DCT to the downstream K^+^-secreting segments, that is the connecting segment (CNT) and cortical collecting duct (CCD). In this scheme, a drop in serum [K^+^] activates NCC resulting in more Na^+^ reabsorption along the DCT. This reduces delivery of Na^+^ to the CNT/CCD, where Na^+^ entry through the amiloride-sensitive epithelial Na^+^ channel (ENaC) provides the drive for K^+^ secretion through the renal outer medullary K^+^ channel (ROMK). Thus, higher NCC activity tends to increase serum [K^+^] by lowering urinary K^+^ excretion. When serum [K^+^] increases, NCC is inhibited, more Na^+^ reaches ENaC, and thus more K^+^ is secreted through ROMK, providing a mechanism to lower serum [K^+^]. This effect of higher serum [K^+^] to inhibit NCC may contribute to the effect of dietary K^+^ supplementation through salt substitutes to provide a protective effect in cardiovascular disease.^61,62^ This protective effect occurs in part by a reduction in systolic and diastolic BP. An increase in serum [K^+^] as a result of higher K^+^ intake may thus exert a diuretic effect by lowering NCC activity. This is similar to the mechanism by which inhibition of NCC by thiazide diuretics or genetic inactivation of NCC in Gitelman syndrome or Gitelman syndrome-like disorders leads to hypokalemia. Persistent Na^+^ delivery to the CNT/CCD occurs regardless of a decrease in serum [K^+^].

The molecular basis of the serum [K^+^] modulation of NCC activation involves both the WNK-SPAK/OSR1 pathway and phosphatases. WNK kinases directly bind Cl^−^, which inhibits their activity^63^ (Figure 1). A reduction in serum [K^+^] promotes efflux of Cl^−^ from DCT cells activating WNK4 and hence SPAK and NCC. When serum [K^+^] increases, higher intracellular Cl^−^ inhibits WNK4-SPAK/OSR1 signaling,^64^ which coupled with phosphatase activity leads to a lower degree of NCC phosphorylation and activity (Figure 2). In FHHt, NCC cannot be switched off despite a rise in serum [K^+^] because the causative mutations in *WNK1*, *WNK4*, *CUL3*, or *KLHL3* lead to high levels of WNK kinases. This overrides the high serum [K^+^] signal, causing persistent hyperkalemia, although there may be some more direct effects of the mutant proteins on ENaC and ROMK.^38,65^

### Other Signaling Pathways

Hypertension in the insulin-resistant state and in hyperinsulinemia is primarily mediated by the insulin-induced stimulation of renal reabsorption of Na^+^.^66,67^ In support of this, Bickel and colleagues reported that obese Zucker rats with hyperinsulinemia showed increased abundance of NCC and hypertension resulting from Na^+^ retention.^68^ Insulin-induced stimulation of NCC activity has been shown to be related to activation of the WNK4-SPAK pathway *via* the phosphatidylinositol 3-kinase/Akt signaling pathway both *in vitro* and *in vivo* in obese Zucker rats and the db/db mouse model of hyperinsulemic metabolic syndrome.^69–71^

Walsh and colleagues reported that the combined infusion of NE and a high salt diet resulted in the activation of NCC and salt-sensitive hypertension in rats.^72^ Other studies indicate that NE administration increased abundance of phosphorylated NCC, and BP in mice on both normal and high salt diet.^73,74^ The effect of NE to stimulate NCC phosphorylation was impaired in renal tubule-specific OSR1 knockout mice, suggesting that OSR1 rather than SPAK mediates the effect.^74^

Uromodulin (UMOD), also known as Tamm–Horsfall protein, is a glycoprotein produced not only predominantly by the TAL (85%–90%) but also by the early part of the DCT (10%–15%).^75^ While a large portion of UMOD synthesized by the TAL is released into the tubular lumen and interstitium, intracellular UMOD regulates protein trafficking and sorting and organizes lipid microdomains of the apical membrane *via* its GPI-anchoring site.^76^ UMOD knockout mice displayed a decrease in phosphorylation of NCC along the early part of the DCT that was compensated for by increase in the phosphorylation of NCC in the later part of the DCT.^75^ A reduction in phosphorylated SPAK/OSR1 along the early DCT in UMOD knockout mice suggests involvement of the WNK-SPAK/OSR1 pathway in activation of NCC by UMOD. Two studies in humans have shown that UMOD may play a significant role in BP regulation in humans. A genome-wide association study revealed that common variants in the *UMOD* promoter associated with lower UMOD expression are also associated with a reduced risk of arterial hypertension and cardiovascular events.^77^ Individuals with *UMOD* promoter variants associated with higher expression of UMOD displayed an enhanced response to furosemide with respect to BP reduction.^78^ Whether higher NCC activity contributes to the hypertensive effects of *UMOD* promoter variants that raise BP has not been determined.

Other pathways that have been demonstrated to activate NCC through WNK-SPAK/OSR1 signaling in experimental models include vasopressin,^79^ parathyroid hormone,^80^ and the calcium-sensing receptor^81^; how activation of NCC by these relate to changes in BP is unknown. Activating pathways are summarized in Figure 1.

## Phosphatases Inactivate NCC Both Directly and Indirectly

Counteracting activation by WNK-SPAK/OSR1-dependent phosphorylation, phosphatases remove amino-terminal phosphates from NCC directly and from the activation loops of WNK kinases and SPAK/OSR1, switching off their kinase activity (Figure 2). There is less clarity regarding the specific phosphatases involved. There is evidence for direct and indirect effects of several including protein phosphatase 1 (PP1), protein phosphatase 2A, calcineurin (CN), and protein phosphatase 4 but *in vivo* evidence for roles for protein phosphatase 2A and protein phosphatase 4 is lacking (for a recent in-depth review see ref. 82). PP1 may directly dephosphorylate NCC^83,84^ and may also indirectly lower NCC phosphorylation by dephosphorylating WNK4^85^ and SPAK/OSR1.^86^ PP1 is inhibited by I1, a regulatory subunit. Importantly, I1 knockout mice with lower PP1 inhibition display lower levels of phosphorylated NCC and lower BP.^83^

CN is a phosphatase classically inhibited to provide immunosuppression in transplant patients. In a subset of patients receiving the CN inhibitors, side effects closely resembling FHHt are observed, including hypertension, hyperkalemia, and metabolic acidosis. Administration of the CN inhibitors tacrolimus (also known as FK506) to mice^87^ or cyclosporine^88^ to rats causes similar effects, with an associated increase in NCC phosphorylation. The effect of tacrolimus to increase BP was absent in NCC knockout mice or with thiazide administration. Higher abundances of both total and phosphorylated NCC have also been observed in urinary vesicles isolated from patients treated with CN inhibitors.^89^ A prospective study revealed positive correlation between total and phosphorylated NCC abundances and the development of hypertension in a cohort of male patients undergoing kidney transplant.^90^ Furthermore, higher abundances of total and phosphorylated NCC correlated with thiazide responsiveness in a cohort of hypertensive kidney transplant recipients.^89^ However, in a randomized noninferiority crossover trial, the thiazide chlorthalidone was shown to have a similar BP-lowering effect as the calcium channel blocker amlodipine in hypertensive kidney transplant recipients.^91^ CN is not likely to directly dephosphorylate NCC under baseline conditions but has been reported to impair NCC dephosphorylation following acute K^+^ loading, without affecting phosphorylation of SPAK.^92^ Studies with CN inhibitors have also shown increased abundances of WNK kinases^87,88^ and total and phosphorylated SPAK/OSR1^87,93^ (Figure 2). The actual target of CN may be KLHL3 because KLHL3 phosphorylation was higher in cells treated with tacrolimus and in mice with tacrolimus-induced hypertension.^93^ As noted above, KLHL3 phosphorylation impairs WNK4 binding to CUL3-KLHL3, preventing WNK4 ubiquitination and subsequent proteasomal degradation, enhancing WNK-SPAK/OSR1-mediated NCC phosphorylation.

## Conclusion

Since the discovery of WNK kinase mutations as causing FHHt, a complex pathway for NCC regulation involving modulation of NCC phosphorylation has been elucidated. The identification of additional causative mutations in *CUL3* and *KLHL3* revealed a further layer of regulation. Many animal models have confirmed that the CUL3/KLHL3–WNK-SPAK/OSR1 pathway plays a critical role in modulating NCC activity with consequences on BP. Increased activity of NCC results from phosphorylation-induced activation mediated by the WNK-SPAK/OSR1 kinases, whereas lower NCC activity is mediated through ubiquitination by of the WNK kinases by the CUL3-KLHL3 E3 ubiquitin ligase complex. Phosphatases such as CN also modulate activity of the pathway, switching it off by lowering kinase activity of WNK-SPAK/OSR1 or by promoting WNK4 degradation by dephosphorylating KLHL3. Ultimately, the combined effects of these regulators on NCC directly affect BP. In the future, novel diuretics under development that target WNK kinases and SPAK to inhibit NCC indirectly may become clinically available to treat hypertension.^94–96^
